# Psychooncological distress in low-grade glioma patients—a monocentric study

**DOI:** 10.1007/s00701-021-04863-7

**Published:** 2021-06-22

**Authors:** Alessandra Ley, Marcel Kamp, Christiane von Sass, Daniel Hänggi, Michael Sabel, Marion Rapp

**Affiliations:** Department of Neurosurgery, Heinrich-Heine-University, Moorenstr. 5, 40225 Duesseldorf, Germany

**Keywords:** Low-grade glioma, Distress, HADS, Po-Bado, DT

## Abstract

**Background:**

Patients diagnosed with low-grade glioma (LGG) must live with constant knowledge of an upcoming malignant tumor transformation which may lead to increased anxiety and reduced quality of life. Here, we (1) analyzed the prevalence and risk factors for distress in LGG patients using (2) different screening tools to subsequently (3) evaluate their need for psychological support.

**Method:**

Patients with LGG-suspicious findings in MRI studies as well as patients with histopathological confirmed LGG were screened using three established self-assessment instruments (Hospital Anxiety and Depression Scale, Distress Thermometer, EORTC-QLQ-C30-BN20). Screening results were correlated with sociodemographic factors.

**Results:**

One hundred forty-nine patients (74 men and 75 women) were prospectively included. Patients were further divided into different subgroups regarding the time of screening and diagnosis. An increased level of distress was observed in 20.8% (mean score 1.21, 95% CI 1.15–1.28) of all patients screened by HADS. Significant associated factors were pre-existing psychiatric disorders (*p* = 0.003) and psychotropic medication (*p* = 0.029). HRQoL (*p* = 0.022) and global health item (*p* = 0.015), as well as future uncertainty (*p* = 0.047), assessed by the EORTC-QLQ-C30-BN20 were significantly higher in those patients without histopathological diagnosis. Increased distress was significantly correlated with results in chosen sub-items of the HRQoL questionnaire (*p* < 0.001).

**Conclusions:**

Our results demonstrate the need for frequent distress screening. If specific tools are not available, HRQoL questionnaires can also be used. Patients with pre-existing psychological stress should be offered additional psychooncological support, irrespectively of the time of screening or tumor diagnosis.

*Clinical trial registration number:* 4087

## Introduction

Brain tumor patients are at high risk of suffering from psychooncological distress [5], which may cause deterioration of health-related quality of life (HRQoL) [21] and even lead to decreased overall survival [23]. Although one-third of all cancer patients experience high levels of distress with the need of special support [8] [4], most are unaware that additional psychooncological treatment is available and accessible [8]. Therefore, elevated distress often remains undetected and untreated [8]. However, early psychooncological intervention can enhance the patient’s HRQoL and medical outcome before distress and depression have a negative impact on functional status and quality of life [37]. Therefore, consistent screening is indispensable. However, existing screening instruments are rarely used in clinical daily routine [26] especially in a neurosurgical environment.

Among brain tumor patients, those with low-grade gliomas (LGG) represent a unique subset. Patients leading a normal life are faced with a tumor which will inevitably migrate along with the white matter and progress to high-grade glioma (HGG) [11]. Clinical symptoms (i.e., epileptic seizures) can lead to dramatic obligatory changes in lifestyle and daily routines. Patients and their families are confronted with increasing cognitive and neurological deficits. However, the diagnosis of LGG is generally associated with favorable survival time [16]. This means that the knowledge of a certain malignant tumor transformation [16] and the incurability of this disease with only contemporary medical treatment options [11] are a constant companion.

This disease-specific burden is poorly represented in existing studies since literature regarding supportive needs of brain tumor patients mostly concern patients with HGG. If considered, LGG patients are included in small numbers or analyzed together with different benign tumor entities [9, 10, 22, 24, 25, 36, 39]. Therefore, little is known about the disease-specific burdens and prevalence and clinical significance of depressive and anxiety symptoms in LGG patients. Due to these specific characteristics, a separate evaluation of LGG patients concerning their distress and HRQoL is essential [11] in order to understand and address their disease-specific needs and to identify important impact factors for increased distress.

Here, we investigated the psychooncological distress of LGG patients during the course of therapy. Due to individual treatment decisions, some LGG are only diagnosed via MRI without a histopathological confirmation. Do these patients need special support because of this “unknown threat” or—in contrast—do these patients maybe cope better with this situation with respect to other LGG patients with confirmed histopathology?

Concluding, the purpose of this study was (1) to analyze the prevalence and clinical risk factors of distress and depression in LGG patients at different time-points of their disease, (2) to compare different screening methods regarding their clinical use, and to finally (3) evaluate the need for psychological support.

## Patients and methods

### Ethical statement

The study was approved by the local ethics committee (study number 4087). All patients obtained informed consent. The withdrawal was possible at any time on the patient’s request.

### Study design and patients

Between October 2013 and January 2016 LGG patients, who were treated at the department of neurosurgery of the University Hospital Duesseldorf, were prospectively included. Patients were screened for their psychological distress using two self-assessment instruments (Hospital Anxiety and Depression Scale (HADS) and Distress Thermometer (DT)). Sociodemographic factors were screened by the external assessment questionnaire (Psychooncological base documentation (PO-Bado)). Additionally, HRQoL was assessed using the EORTC-QLQ-C30-BN20 questionnaire.

Inclusion criteria for this study were patients (1) with the neuropathological diagnosis of a WHO grade II LGG or (2) with an untreated cerebral lesion suspicious for WHO grade II LGG on MRI who were (3) treated at the neurosurgical- neurooncological center of the university hospital of Duesseldorf and (4) were screened for their psychooncological distress as described below in the course of their illness. All patients did not receive any other therapy (chemotherapy or radiation) other than an operation. In our clinical practice, a watchful waiting strategy is used in patients with suspected grade II lesions with eloquent localization of the tumor and negative FET-PET-MRI or in those patients who refuse surgical approach.

Patients (1) under the age of 18, (2) with a severe aphasic disorder, (3) lacking the ability to give consent, (4) under palliative care, (5) with physical or cognitive inability to complete the screening instruments, and (6) patients who underwent tumor-malignization or progress were excluded from this study.

Inpatient screening was performed after surgery, outpatient screening during scheduled consultations for routine MRI scans. Further patient characteristics are summarized in Table 1.

**Table 1:** Patients’ characteristics and demographic data; *p* value of group differences (Group A–C) analyzed by Chi-square test *NR* not reported, †percent of missing values,-patient number too small for analyses. Group A, screening up to 6 months after diagnosis; Group B, screening between 6 months and 3 years after diagnosis; and Group C, screening from 3 years after initial diagnosis

	All patients (*n* = 149) *n* (%)	Group A (*n* = 52) *n* (%)†	Group B (*n* = 38) *n* (%)†	Group C (*n* = 59) *n* (%)†	Group differences *p* =
Gender					
Male	74 (49.7)	24 (46.2)	23 (60.5)	27 (45.8)	0.3
Female	75 (50.3)	28 (53.8)	15 (39.5)	32 (54.2)	
Mean age (years)	46	44	50	47	0.66
Range	19–84	19–84	20–78	22–75	
Diagnosis					
Suspected LGG	53 (35.6)	19 (36.5)	25 (65.8)	9 (15.3)	-
Diffuse astrocytoma	58 (38.9)	21 (40.4)	6 (15.8)	13 (22.0)	
Oligodendroglioma	22 (14.8)	5 (9.6)	4 (10.5)	6 (10.2)	
Oligoastrocytoma	16 (10.7)	7 (13.5)	3 (7.9)	31 (52.5)	
Relationship status					
Partnership	117 (78.5)	38 (73.1)	32 (84.2)	47 (79.7)	0.5
Single	30 (20.1)	13 (25.0)	6 (15.8)	11 (18.6)	
NR	2 (1.3†)	2 (3.5†)	0	1 (1.7†)	
Children					
Yes	95 (63.8)	30 (57.7)	23 (60.5)	42 (71.2)	0.28
No	52 (34.9)	21 (40.4)	15 (39.5)	16 (27.1)	
NR	2 (1.3†)	1 (1.9†)	0	1 (1.7†)	
H/o psychiatric disorders					
Yes	40 (26.8)	17 (32.7)	15 (39.5)	26 (44.1)	0.47
No	107 (71.8)	34 (4.4)	23 (60.1)	32 (54.2)	
NR	2 (1.3†)	1 (1.9†)	0	1 (1.7†)	
H/o psychotropic meds					
Yes	58 (38.9)	10 (19.2)	11 (28.9)	19 (32.2)	0.294
No	89 (59.7)	41 (78.8)	27 (71.1)	39 (66.1)	
NR	2 (1.3†)	1 (1.9†)	0	1 (1.7†)	

We analyzed the impact of time and the influence of the histopathological confirmation at different time points during the course of therapy. Therefore, patients were further divided into different subgroups:

- Group A: screening up to 6 months after initial diagnosis;
- Group B: screening between 6 months and 3 years after diagnosis and
- Group C: screening from 3 years after diagnosis.
- Group 1: patients with the histopathological confirmation of an LGG,
- Group 2: patients with LGG-suspicious findings in MRI in a watchful-waiting strategy without histopathological confirmation.

The following medical information were further analyzed: age, gender, the status of the disease, neurosurgical diagnosis, social factors like relationship status, children, and occupation. In addition, data regarding pre-existing psychiatric disorders and medication with ataractics were collected.

### Distress screening instruments

In 2014, the German Cancer Society published the S3-guideline, concluding that the Hospital Anxiety and Depression Scale (HADS-D) carried the best evidence even though it was not developed specifically for cancer patients. As an alternative, the Distress Thermometer (DT) was recommended [19].

Based on these recommendations, we selected the HADS and the DT as two self-assessment tools to test for increased distress. In addition, we chose the basic documentation for psychooncology (Po-Bado), as an objective assessment of the patient’s subjective psychosocial condition. Subsequently, we analyzed the screening results and correlated them with clinical and demographic data. Likewise, we correlated the results concerning increased neuropsychological distress with the HRQoL questionnaire (EORTC-QLQ-C30 -B20).

HADS was originally designed to assess the psychological state of physically ill patients. Meanwhile, it has been established as an effective screening tool for the assessment of anxiety and depression [14, 27, 30]. The 14-item self-report questionnaire consists of 7 items used to identify anxiety (HADS-A) and 7 items for depression (HADS-D), with each item having a 4-point (0–3) Likert-type scale. Two thresholds are recommended: ≥ 8 for greater sensitivity and ≥ 11 for greater specificity [1]. We used a cut-off at ≥ 11 to define a pathological HADS-A or HADS-D screening result.

PO-Bado is a semi-directive instrument for assessing psychosocial difficulties which have been designed and validated in Germany [6, 13, 20]; in this study, we used the PoBado to gather information about the patients’ demographics and psychosocial background and to detect patients with the need for psychooncological support, previously described stratification criteria are applied [20].

The DT is a single-item visual analog scale (ranging from 0 (no distress) to 10 (maximum distress)), developed to rapidly screen patients for psychological distress, initially designed by Roth et al. [14, 30, 35, 41] According to the NCCN guidelines, we defined a DT score of 5 or above as indicating distress [27]. The DT also contains a list of 40 symptoms representing practical, family, emotional, spiritual-religious, and physical concerns. In our setting, we only used the visual scale; the symptom list was excluded.

According to Goebel et al., the HADS was used as a gold standard against which the other tests were compared [13]. Independently from their screening results, patients were asked if they wanted a psychooncological consultation.

### Health-related quality of life assessment

The EORTC QLQ-C30-BN20 is a disease-specific questionnaire developed by the European Organization for Research and Treatment of Cancer (EORTC) to assess the quality of life of cancer patients. The EORTC QLQ-C30 consists of a four-point scale containing five function scales, three symptom scales, and six single-item scales as well as two seven-point scales: the global health status and the quality of life. The QLQ-BN20 is an additional module for brain tumor patients, consisting of 20 questions specifically assessing brain tumor-related symptoms [1, 38]. Distress screening results were correlated with the following items: global health status, quality of life, emotional and cognitive function, and future uncertainty [16, 17]. The threshold for the global health and quality of life score was $\le$ 4 and for emotional function, cognitive function, and future uncertainty $\ge$ 2.75 scored according to the recommended scoring manual of the EORTC.

### Statistical analysis

Despite the prospective design of the study, all analyses were descriptive; therefore, results were regarded as hypothesis-generating only. Statistical analyses were performed using the software IBM SPSS Statistics 25.0 (IBM, Armonk, New York, US). Data was described by standard descriptive statistics, using absolute and relative frequencies for categorical variables and median for continuous variables. *T* test and univariate logistic regression modelling were used for categorical and continuous variables when normal distribution was given. The frequency of distribution was further analyzed with the Chi-square test. Before using non-parametric tests (Mann–Whitney *U*, Kruskal–Wallis), we considered data for normal distribution (Kolmogorov–Smirnov test). Sensitivity and specificity were determined using cross-tables. Receiver operator characteristic (ROC) curve analyses were conducted using HADS as the gold standard. The area under the ROC curve (AUC) represents the overall performance of the different screening instruments in their ability to discriminate between patients with and without conspicuous HADS-results. An AUC of 0.7–0.8 reflects fair, of 0.8–0.9 good discrimination. An AUC above 0.9 would reflect excellent discrimination.

In addition, all relations between HADS-A and HADS-D and EORTC items were assessed by multiple linear regression; HADS-A and HADS-D were used as continuous variables. In the regression analyses, HADS was used as a dependent variable. Furthermore, binary logistic regression analyses were performed using EORTC-dimensions (conspicuous vs non-conspicuous) using HADS (conspicuous in HADS-A or HADS-D) as a dependent variable.

Binary logistic regression was used to predict the odds of independent variables working as a predictor on the values. The coefficient of regression (*R*) is only described if significant.

Patients with missing data were excluded from the corresponding statistical analyses but not from the entire study.

A significance was considered clinically relevant when *p* = 0.05. All reported *p* values are two-sided. A confidence interval of 95% was used. All *p* values are corrected *p* values; post hoc tests were included in every calculation.

## Results

### Patients

From 2013 until 2016, 149 patients (74 males (49.7%), 75 females (50.3%), mean age 46 years (range 19–84 years)) took part in this study. In 53 patients (35.6%), the diagnosis of LGG was provided solely via MR imaging (Group 2). In 96 patients (64.4%, Group 1), the diagnosis was histologically confirmed to be diffuse astrocytoma (58 patients, 38.9%), diffuse oligodendroglioma (22 patients, 14.8%), and diffuse oligoastrocytoma (16 patients, 10.7%). During this study period, around 172 patients with LGG were treated; we were able to include 149 (86.6%) patients; 57 patients were operated on an LGG during that time; we were able to screen 80.7% (46 patients) of these.

Depending on their initial time of screening, patients were further divided into three groups. Group A (52 patients) were screened until 6 months after the initial diagnosis (histologically or suspected in imaging). Group B (38 patients) were screened 6 months to 3 years after initial diagnosis. Group C (59 patients) were screened 3 years after receiving their initial diagnosis. Clinical and sociodemographic data, as well as statistical results, are presented in Table 1.

Regarding the different questionnaires, 145 (97.3%) patients completed the HADS, 138 (92.6%) patients completed the DT, and 149 (100%) patients completed the PoBado. The EORTC questionnaire was completed by 124 (83.2%) patients. Complete screening results are illustrated in Table 2.

**Table 2:** Conspicuous screening results assessed by HADS. Univariate analysis (normal distribution), Mann–Whitney *U* and Kruskal–Wallis (normal distribution not given), and Cramer’s *V* were performed in order to compare different subgroups regarding their presence of psychooncological distress. *n* number of patients analyzed. ^a^Hospital Anxiety and Depression Score (*D* depression, *A* anxiety). ^b^*p* value for comparison of screening results of the different assessment tools and different subgroups of patients. Bold printed values indicate significant results (*p* < 0.05)

	HADS-D^a^ ≥ 11 *n* = 143 *n* (%)	*p*^b^	HADS-A^a^ ≥ 11 *n* = 143 *n* (%)	*p*^b^	HADS-D^a^ or HADS-A^a^ ≥ 11 *n* = 145 *n* (%)	*p*^b^
All patients	24 (16.1)		24 (16.1)		31 (20.8)	
Male	11 (12.8)	0.64	9 (10.5)	** < 0.001**	13 (15.1)	0.12
Female	14 (17.7)		16 (20.3)		19 (24.1)	
Age < 65	24 (16.1)	0.85	23 (15.4)	0.18	30 (20.1)	0.58
Age > 65	1 (6.7)		2 (13.3)		2 (13.3)	
Partnership	18 (14.4)	0.23	19 (15.2)	0.26	24 (19.2)	0.63
No partnership	7 (18.9)		6 (16.2)		8 (21.6)	
Children	18 (17.8)	**0.023**	18 (17.8)	**0.044**	24 (23.8)	0.1
No children	7 (11.5)		7 (11.5)		8 (13.1)	
Pre-existing psychiatric disorders						
Yes	14 (23.3)	** < 0.001**	14 (23.3)	** < 0.001**	19 (31.7)	**0.003**
No	11 (10.9)		11 (10.9)		13 (12.9)	
Ataractics						
Yes	10 (23.3)	** < 0.001**	9 (20.9)	**0.002**	13 (30.2)	**0.029**
No	15 (12.7)		16 (13.6)		19 (16.1)	

### Hospital Anxiety and Depression Scale (HADS)

In 24 (16.1%, mean score 4.67, 95% CI = 3.85–7.4) patients, the HADS depression score (HADS-D) was increased. Having children (*p* = 0.023), pre-existing psychiatric disorders (*p* $\le$ 0.001) and a history of antidepressant drugs (*p* $\le$ 0.001) were associated with higher HADS-D screening results (Table 2).

Regarding the HADS anxiety score (HADS-A), increased scores were observed in 24 patients (16.1%, mean score 5.73, 95% CI = 4.98–6.48). Notably, female patients (*n* = 16, 20.3%, *p* $\le$ 0.001), patients with children (*p* = 0.044) patients with pre-existing psychiatric disorders (*p* $\le$ 0.001), and patients with a history of anti-depressant drugs (*p* = 0.002) demonstrated increased scores at significant levels (Table 2).

In addition, we assessed the HADS-T (combined scores of HADS-D and HADS-A), i.e., scores of $\ge$ 11 for HADS-D and/or HADS-A (Table 2). Similar to the single score evaluation, patients with pre-existing psychiatric disorders (*p* = 0.003) and a history of antidepressant drugs (*p* = 0.029) were significantly more likely to experience increased distress (Table 2). Nineteen patients (34.5%) with a history of mental disorders presented with conspicuous HADS results whereas 12 (13.6%) patients without prior history showed higher depressive and anxiety scores (*p* = 0.003).

### Distress Thermometer (DT)

More than half of patients (*n* = 84, 56.4%, mean score 5.12, 95% CI = 4.66–5.58) marked a DT score above the threshold of 5. There were no factors which correlated significantly with increased scores. There was no significant difference between the patient groups.

### Psychooncological base documentation (PoBado)

Screening with PoBado revealed that 36 (24.2%, mean score 1.24, 95% CI = 1.17–1.31) patients described the subjective need for further psychooncological support. There was no significant difference between the patient groups (Group A, B, C: *p* = 0.77; Group 1, 2: *p* = 0.26).

### EORTC-QLQ-C30-BN20

**Fig. 1:**
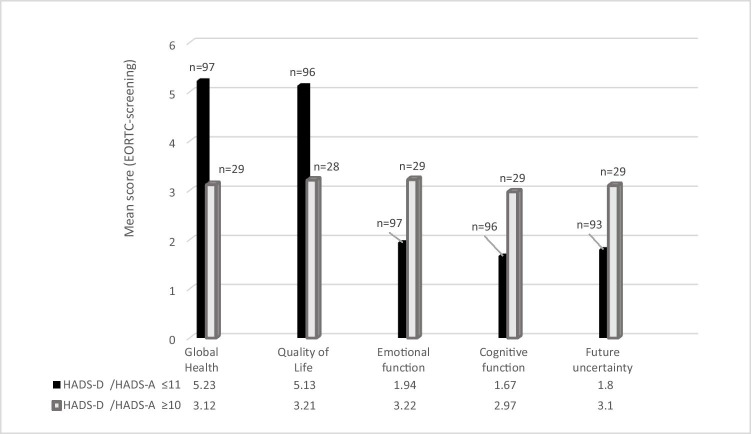
Block-diagram presenting EORTC-QLQ-C30-BN20 subscales results (presented as mean scores) and their correlation to the Hospital Anxiety and Depression Scale (HADS-T) results, statistical test used: Mann–Whitney *U*. Threshold for conspicuous screening concerning the global health and quality of life score was $\le$ 4 and for emotional function, cognitive function and future uncertainty $\ge$ 2.75 scored according to the recommended scoring manual of the EORTC

Considering Groups A, B, and C, there were no significant differences regarding the EORTC-QLQ-C30–BN20 items (global health status *p* = 0.74, quality of life *p* = 0.72, emotional function *p* = 0.77, cognitive function *p* = 0.08, future uncertainty *p* = 0.96). All EORTC-items mean scores had a significant correlation to higher HADS-scores (*p* =  < 0.001 for every item, statistical test used: Mann–Whitney *U*, results are presented in Fig. 1).

Regression analyses emphasize these connection; multiple linear regression results can be found in Table 3. Linear regression analyses show that especially high future uncertainty (*p* = 0.003) and higher emotional function deficits (*p* = 0.001) can explain high scores in HADS-A with a security of 53.6% (Table 3), and low global health (*p* = 0.014), high future uncertainty (*p* = 0.013), and decreased cognitive function (*p* = 0.001) can predict higher scores in HADS-D with a security of 59.9% (Table 3). Binary logistic regression analyses indicate that higher emotional function deficits (*p* = 0.012, regressions coefficient (*R*) = 1.54) and higher future uncertainty (*p* = 0.004, *R* = 1.59) can be used as predictors for elevated distress.

**Table 3:** Multiple linear regression analyses of HADS-A and HADS-D and EORTC items

EORTC-item	Unstandardized	Standardized	Standard error	*p* =
Dependent variable: HADS-A				
Constant	0.71		2.184	
Quality of life	− 0.112	− 0.038	0.405	0.782
Global health	− 0.321	− 0.115	0.383	0.4
Future uncertainty	1.432	0.281	0.478	**0.003**
Emotional function	1.7323	0.353	0.527	**0.001**
Cognitive function	0.297	0.65	0.427	0.488
* R*^2^	0.556			
Adjusted *R*^2^	0.536			
* F*	27.78			
Dependent variable: HADS-D				
Constant	3.718		2.348	
Quality of life	− 0.064	− 0.019	0.42	0.879
Global health	− 1.003	− 0.31	0.403	**0.014**
Future uncertainty	1.278	0.219	0.507	**0.013**
Emotional function	0.335	0.059	0.561	0.551
Cognitive function	1.642	0.312	0.459	**0.001**
* R*^2^	0.616			
Adjusted *R*^2^	0.599			
* F* (df = 5; 128)	36.01			

Considering separation into Groups 1 and 2, the QoL item (*p* = 0.022) and global health item (*p* = 0.015), as well as future uncertainty (*p* = 0.047), were significantly higher in those patients without histopathological diagnosis. There were no other significant differences between the groups concerning the other items (emotional function *p* = 0.61, cognitive function *p* = 0.43).

### Sensitivity and specificity

**Fig. 2:**
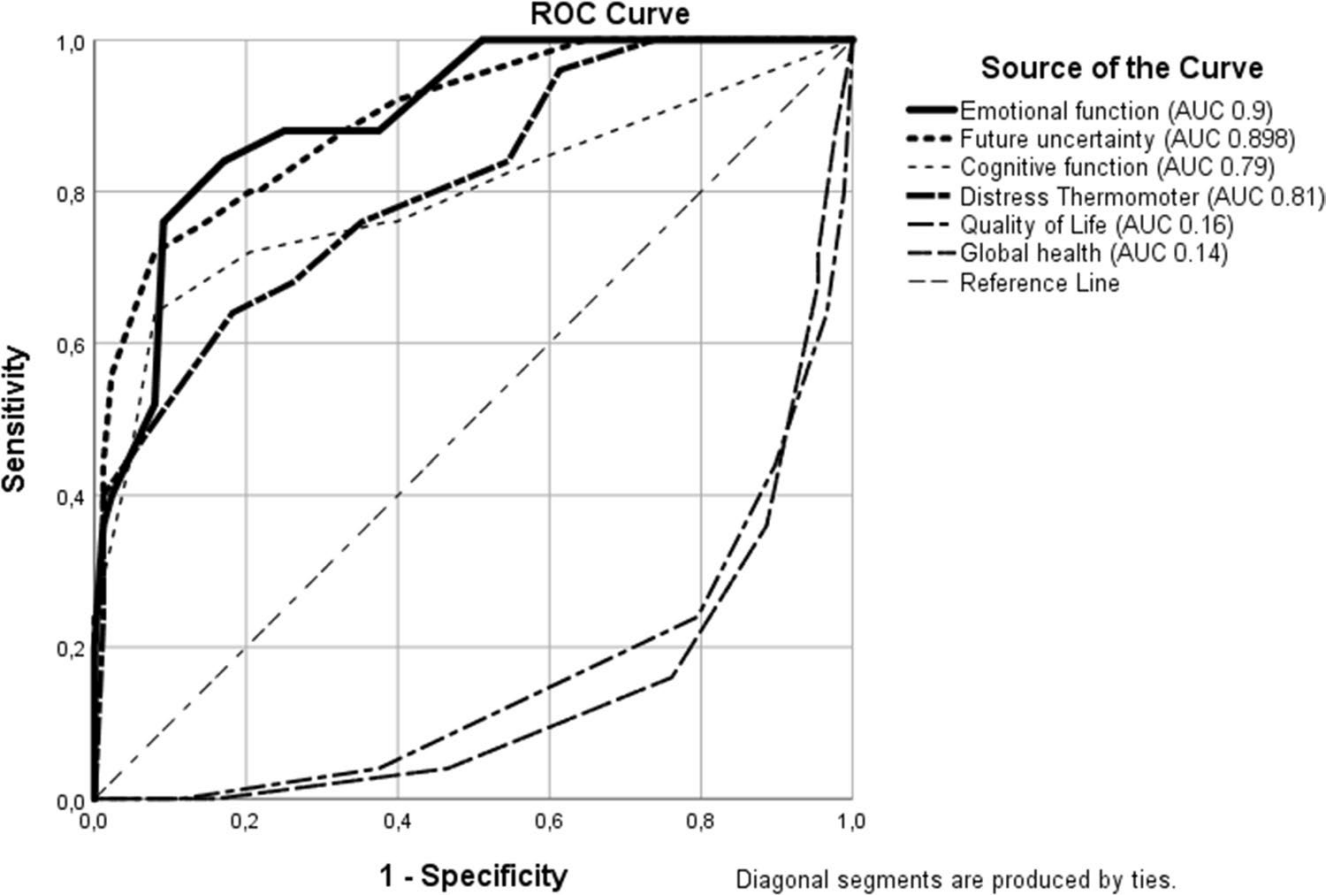
ROC analyses were performed using the HADS as gold-standard against the other screening instruments. The area under the curve reflects the conspicuous screening results using the other tests compared to conspicuous and non- conspicuous results in the HADS. An AUC between 0.7 and 0.8 reflects fair discrimination results, between 0.8 and 0.9 reflects a good discrimination

Screening results of the different assessment tools differed widely. While the HADS-T indicated increased distress in 31 patients (20.8%), the DT showed scores ≥ 5 in 84 patients (56.4%) revealing a sensitivity of 84.6% and a specificity of 45.0%. ROC curves can be found in Fig. 2. Increased HADS screening results were furthermore correlated to specific items of the HRQoL assessment regarding sensitivity and specificity. The quality of life item hereby demonstrated a sensitivity of 75.0% and a specificity of 79.4%, the global health item demonstrated a sensitivity of 82.8% and a specificity of 77.1%, the future uncertainty item demonstrated a sensitivity of 69.0% and a specificity of 91.4%, the emotional function item demonstrated a sensitivity of 75.9% and a specificity of 88.7%, and the cognitive function item demonstrated a sensitivity of 69% and a specificity of 91.7%.

### Need for psychooncological support

Independently from the distress screening results, 33 patients (22.8%, Group A: *n* = 13, Group B: *n* = 8; Group C: *n* = 13) asked for further psychooncological support; however, 23 of these patients (69.7%, screened by HADS) presented with a negative screening result. Results assessed by the HRQoL questionnaire demonstrated a significant correlation with the demand for psychosocial support with decreased cognitive function (*p* = 0.038).

## Discussion

LGG patients represent a specific patient cohort due to the unique problems they are facing in their daily life caused by the diagnosis itself, tumor progression, and/or therapy (side) effects. However, these disease-specific impacts on distress, depression, and HRQoL are not routinely assessed. The purpose of this study was to evaluate LGG patients’ distress to identify disease-associated risk factors in order to address supportive care needs and finding a suitable screening mechanism. Secondly, we analyzed if there are any differences in the level of distress in patients who have a histopathological confirmed diagnosis as opposed to those under watchful waiting. Furthermore, we investigated if psychooncological distress levels differed at different times in the course of the disease from postoperatively to years after diagnosis. To our knowledge, this is the largest series of LGG patients analyzed with respect to their psychooncological distress.

The impact of brain tumor diagnosis with respect to its entity on psychooncological distress still remains controversial. Armstrong hypothesized that the diagnosis of a brain tumor itself is responsible for the patients’ depressive symptoms regardless of the histological diagnosis [3]. Keir pointed out that LGG patients reported higher levels of stress compared to HGG patients [18] in contrast to Bunevicius observed higher scores on depression scales in HGG patients [6]. Mainio however found that depression had a long-term effect on survival in LGG patients [25], which might be explained by the differing survival probability. However, all the abovementioned studies included only small patient numbers, which might be an explanation for the contradicting findings. In this study, we analyzed a much larger patient number. Here, no significant differences concerning distress in patients with a histologically confirmed diagnosis of LGG and those without a definite diagnosis were observed.

### Depression and anxiety assessment in LGG patients

When assessed using the HADS, depression in brain tumor patients reaches a prevalence between 15 and 38%. Some authors hypothesized a fluctuation during the course of therapy with an increasing depression rate throughout the course of the disease [9, 20, 23]. Goebel and Mehdorn could not find a correlation between depression and time of screening, but increased anxiety rates in inpatient screening [13]. In this study, we observed increased depression and anxiety in 16.1% of patients independently from time of screening and setting (inpatient or outpatient, Table 2). This finding might indicate that LGG patients suffer from depression right after receiving the initial diagnosis and that anxiety remains a constant companion.

### Distress assessment of LGG patients

Bunevicius estimates that 18–22% [5] of brain tumor patients suffer from moderate to severe distress. Our study found a similar incidence of distress (20.8% of patients, Table 2). Goebel described higher levels of distress after surgical interventions when compared to follow-up visits [13], while our findings suggest that distress rates are independent from the time of screening. Trad describes higher distress levels in HGG patients compared to other tumors, but only 8.3% of his patients suffered from LGG [40]. Referring to our results including preliminary studies of our group [31], however, it is likely that distress is as present in LGG patients as it is in HGG patients. Even so, what still needs to be clarified is how distress develops over the course of time in LGG and how it differs from other tumor entities.

### Disease-specific risk factors in LGG patients

In this study, the leading risk factors for developing increased distress were a history of (or current) psychiatric disorders and psychotropic medication. Similar factors have been described by Mainio, who found that 74% of patients with depression also had a history of depressive episodes [24]. Furthermore, D’Angelo observed an increased level of in patients with current depression [9]. Concerning anxiety, in this study, women tended to report higher rates of anxiety when compared to men. Existing studies report varying results on gender-related differences [9, 13]. Having children represents an additional independent risk factor for increased depression concordant with Randazzo, who described increased stress in female patients, patients having children, and patients with a history of psychiatric disorders [29].

All in all, this indicates that especially LGG are at risk to experience distress because of their active and ongoing participation in work- and family life. Special attention should be paid to patients with psychiatric comorbidities.

### Correlation of distress and HRQoL

In the framework of clinical studies, the evaluation of HRQoL is getting more and more important as an objective criterion to estimate the effect of different therapeutic approaches. It is known that increased distress influences HRQoL negatively [17] [15, 29]. Therefore, it is essential to know if this impact is also reflected vice versa. Different studies describe a correlation between specific HRQoL items and distress assessment [15, 17]. Based on this preliminary work, we could clearly demonstrate a significant correlation (Fig. 1). These results are in accordance with Hickmann’s, who demonstrated emotional and cognitive function to distinguish most accurately between increased and normal distress [16]. Depression was further on reported to be associated with poorer HRQoL [28]. Our results go in line with Aaronson as well, who presented the largest study on long-term HRQoL in LGG patients and noted that patients with LGG reported consistently poorer generic HRQL [2]. Aaronson described factors that could be related to decreased HRQoL were female sex, epilepsy burden, and neurocognitive deficits [2], those are comparable to the factors associated to distress in this study (Table 2, Fig. 1). This indicates that maintaining a good HRQoL might positively influence distress and its consequences in LGG patients and should therefore be addressed in psychosocial support.

In addition to that, vulnerability to decreased HRQoL can be associated with personality-related factors. Bunevicius included personality traits in one of his studies concerning health-related quality of life [7]. Of the analyzed factors (extraversion, agreeableness, consciousness, emotional stability, and openness), the factors that could significantly be correlated with higher QoL were emotional stability and consciousness [7]. The screening methods we used do not cover personality traits, so that consciousness is not represented within this study. Nonetheless, emotional stability is reflected by the EORTC item emotional function which is analyzed in this study as well. Still, further studies to reflect the influence of personality traits on distress in LGG patients should be thought of.

### Screening tools assessment

When comparing the conspicuous screening results, the different tools used in this analysis reflect a high variability. Using the HADS as gold standard, we attempted to sort out which one of these screening instruments can be used as an alternative for HADS. Regarding sensitivity (SEN) and specificity (SPE), the selected EORTC items showed higher results than DT (SEN 84.6%, SPE 45.0%). The EORTC-QLQ-C30-BN20 represented the highest SEN and SPE were demonstrated by the emotional function item (SEN 75.9%, SPE 88.7%). ROC analyses (Fig. 2) reflect the best discrimination between distressed and non-distressed patients in emotional function (AUC = 0.9) and future uncertainty (AUC = 0.898).

Regarding these results, we could clearly demonstrate a correlation between increased distress and conspicuous results as assessed by the EORTC QLQ-30-BN20 questionnaire [17] (Fig. 1) and denote the EORTC to be a reliable screening tool to reflect psychooncological distress in patients with LGG.

The DT is a quick, less time-consuming assessment tool with high sensitivity for distress [33]. However, its specificity results rank lower compared to all EORTC-items but with higher AUC-results (Fig. 2). These results are comparable to previous studies, in which the DT has been described as inferior to HADS [34]. In another study, our workgroup developed a screening algorithm that increases the DT’s sensitivity and specificity but only when including the symptom list [31], which in turn reduces the simplicity of this tool.

### Demand for further psychooncological support in LGG patients

In this study, 22.8% of patients expressed their subjective need for further psychological support. Fischbeck described three-quarters of patients suffering from glioblastoma have a psychosocial treatment demand [12]. Renonvanz reported distress to be the most consistent factor associated with the need for further support in brain tumor patients [32]. In her study, the most abundant unmet needs belonged to the psychological domain. Future uncertainty stood out to be the most frequently reported unmet need in high-grade glioma [32]. Other studies reported higher distress to be associated with higher need for support [15]. Here, two significant factors, cognitive and emotional function, could be correlated to higher distress (Fig. 1) and to a demand for psychosocial support. Cognitive and emotional function deficits are not frequently reported in LGG but can be burdensome and have been shown to be the most striking factor to compromise quality of life [39].

### Limitations

Even though this is a prospective study, our patient cohort is quite heterogeneous regarding age, screening time point, and further neurooncological therapy schemes. Patients were screened at different times in the course of their disease independently from their therapy. The impact of radio- or chemotherapy could not be considered because of the patient numbers. In our opinion, this heterogeneity is the most striking limitation but also marks a decisive strength of this data which in the end represents a realistic LGG patient cohort, treated by a neurooncological center.

Another limitation is the WHO classification used in this study. Molecular biomarkers were not available in our patient sample and study; as the data was collected before the WHO classification 2016 was published, we used the WHO classification from 2007. However, we intended to represent all grade II glioma patients regardless of their individual histopathological diagnosis (and molecular markers) to call general attention to the psychosocial distress of all these patients, which, regarding the results of our study, seems to affect not only those with histopathologically known diagnosis but also those without. This leads to the assumption that biological markers might play a secondary role when it comes to psychosocial distress.

### Clinical implications

Our data underline that a multimodal therapy concept that includes psychosocial supportive care is necessary for all glioma patients including LGG patients and regular psychological assessment is indispensable. The presence of psychosocial distress, as well as the demand for psychooncological treatment, is present throughout the whole course of the disease and does not seem to be decreased during the time.

## Conclusion

In conclusion, our data emphasize the importance of psychosocial assessment in clinical daily routine to offer these patients psychooncological support, address anxiety and depression, and improve HRQoL. However, it does not seem to make a difference if the patient is treated surgically or with a watchful-waiting policy. Hereby, special attention should be directed to those with a history of psychological diseases and psychotropic medication. Chosen EORTC-QLQ-C30-BN20 items are suitable to reflect increased distress if specific assessment tools are missing. They can therefore be used to specifically address those patients with conspicuous screening results to detect the need for further psychosocial support.

## Abbreviations

DT: Distress Thermometer
EORTC: European Organization for Research and Treatment of Cancer
HADS: Hospital Anxiety and Depression Scale
HGG: High-grade glioma
HRQoL: Health-related quality of life
LGG: Low-grade glioma
MRI: Magnetic resonance imaging
PO-Bado: Psychooncological base documentation
SEN: Sensitivity
SPE: Specificity
