# Correction to: Psychooncological distress in low-grade glioma patients-a monocentric study

**DOI:** 10.1007/s00701-024-06316-3

**Published:** 2024-10-28

**Authors:** Alessandra Ley, Marcel Kamp, Christiane von Sass, Daniel Hänggi, Michael Sabel, Marion Rapp

**Affiliations:** Department of Neurosurgery, Heinrich-Heine-University, Moorenstr. 5, 40225 Duesseldorf, Germany

**Correction to: Acta Neurochirurgica (2022) 164:713–722**

10.1007/s00701-021-04863-7

The authors regret that we have noted in our recently published report entitled “*Psychooncological distress in low-grade glioma patients-a monocentric study* “ that we inadvertently published tables with calculation errors in table 1 and 2 and concerning the HADS results as well as an indeterminate statement that needs to be clarified. Minor changes concerning the percentage distribution have been made.

These errors do not affect the results and do not alter any statements or conclusions. The discussion and conclusion remain valid.

However, this led to a few clarifying corrections in the body of the manuscript as follows:

In the Abstract, *Results* section, line 3 and 4 should read: An increased level of distress was observed in 22.1% (mean score 1.21, 95% CI 1.15–1.28) of all patients screened by HADS. Significant associated factors were pre-existing psychiatric disorders (p 0.003) and a lack of intake of psychotropic medication (p 0.029).

In the Results section, the *Hospital Anxiety and Depression Scale (HADS)* part should read: In 25 (17.4 %, mean score 4.67, 95% CI=3.85-7.4) patients the HADS depression score (HADS-D) was increased. Having children (p = 0.023), pre-existing psychiatric disorders (p ≤ 0.001) and a lacking history of antidepressant drugs (p ≤ 0.001) were associated with higher HADS-D screening results (table 2).

Regarding the HADS anxiety score (HADS-A), increased scores were observed in 25 patients (17.4 %, mean score 5.73, 95% CI=4.98-6.48). Notably, female patients (n = 16, 20.3 %, p ≤ 0.001), patients with children (p=0.044) patients with pre-existing psychiatric disorders (p ≤ 0.001) and patients with a lacking history of anti-depressant drugs (p = 0.002) demonstrated increased scores at significant levels (table 2).

In addition, we assessed the HADS-T (combined scores of HADS-D and HADS-A), i.e. scores of ≥ 11 for HADS-D and/or HADS-A (table 2). Similar to the single score evaluation, patients with pre-existing psychiatric disorders (p = 0.003) and lacking history of antidepressant drugs (p = 0.029) were significantly more likely to experience increased distress (table 2). 19 patients (13.1%) with a history of mental disorders presented with conspicuous HADS results whereas 12 (8.3%) patients without prior history showed higher depressive and anxiety scores (p=0.003).

In the Results section, *Sensitivity and specificity* part, line* 2* should read*:* While the HADS-T indicated increased distress in 32 patients (22.1 %), the DT showed scores ≥5 in 84 patients (56.4 %) revealing a sensitivity of 84.6 % and a specificity of 45.0 %.

In the Discussion section, *Depression and anxiety assessment in LGG patients* part, line 7 should read: In this study, we observed increased depression and anxiety in 17.4 % of patients independently from time of screening and setting (inpatient or outpatient, table 2).

In the Discussion section, *Distress assessment of LGG patients* part, line 2 should read: Our study found a similar incidence of distress (22.1% of patients, table 2).

In the Discussion section, *Disease‑specific risk factors in LGG patients* part, the first line should read: In this study, the leading risk factors for developing increased distress were a history of (or current) psychiatric disorders and a lack of intake of psychotropic medication.

**Table 1**

In table 1, the last two lines have unintentionally been swapped. As this table contains only descriptive data and the changed results are non-significant, this does not change any declarations made in the article. Table 1 hence needs to read:

**Table 1:** Patients’ characteristics and demographic data; p-value of group differences (Group A-C) analysed by Chi-square-test. NR=not reported, † percent of missing values, - patient number too small for analyses.

Group A: screening up to 6 months after diagnosis;

Group B: screening between 6 months and 3 years after diagnosis and Group C: screening from 3 years after initial diagnosis

**Table:** 

	**All patients** **(n = 149)** **n (%)**	**Group A** **(n = 52)** **n (%)**†	**Group B** **(n = 38)** **n (%)**†	**Group C** **(n =59)** **n (%)**†	**group differences** **p=**
**Gender**					
**Male**	74 (49.7)	24 (46.2)	23 (60.5)	27 (45.8)	0.3
**Female**	75 (50.3)	28 (53.8)	15 (39.5)	32 (54.2)	
**Mean age (years)**	46	44	50	47	0.66
**Range**	19-84	19-84	20-78	22-75	
**Diagnosis**					
**Suspected LGG**	53 (35.6)	19 (36.5)	25 (65.8)	9 (15.3)	**-**
**Diffuse astrocytoma**	58 (38.9)	21 (40.4)	6 (15.8)	13 (22.0)	
**Oligodendroglioma**	22 (14.8)	5 (9.6)	4 (10.5)	6 (10.2)	
**Oligoastrocytoma**	16 (10.7)	7 (13.5)	3 (7.9)	31 (52.5)	
**Relationship status**					
**Partnership**	117 (78.5)	38 (73.1)	32 (84.2)	47 (79.7)	0.5
**Single**	30 (20.1)	13 (25.0)	6 (15.8)	11 (18.6)	
**NR**	2 (1.3†)	2 (3.5†)	0	1 (1.7†)	
**Children**					
**Yes**	95 (63.8)	30 (57.7)	23 (60.5)	42 (71.2)	0.28
**No**	52 (34.9)	21 (40.4)	15 (39.5)	16 (27.1)	
**NR**	2 (1.3†)	1 (1.9†)	0	1 (1.7†)	
**H/o psychotropic meds**					
**Yes**	58 (38.9)	10 (19.2)	11 (28.9)	19 (32.2)	0.29
**No**	89 (59.7)	41 (78.8)	27 (71.1)	39 (66.1)	
**NR**	2 (1.3†)	1 (1.9†)	0	1 (1.7†)	
**H/o psychiatric disorders**					
**Yes**	40 (26.8)	17 (32.7)	15 (39.5)	26 (44.1)	0.47
**No**	107 (71.8)	34 (65.4)	23 (60.5)	32 (54.2)	
**NR**	2 (1.3†)	1 (1.9†)	0	1 (1.7†)	

**Table 2**

In table 2 calculation errors concerning the percentages have been made. The results and significances are hereof unconcerned, as all statistical calculations have been calculated with the integral patient numbers. Table 2 should read:

**Table 2:** Conspicuous screening results assessed by HADS. Univariate analysis (normal distribution), Mann-Whitney U and Kruskal-Wallis (normal distribution not given) and Cramer’s V were performed in order to compare different subgroups regarding their presence of psychooncological distress.

n= number of patients analysed

^a^ Hospital Anxiety and Depression Score (D= Depression, A= Anxiety)

^b^ p-value for comparison of screening results of the different assessment tools and different subgroups of patients. Bold printed values indicate significant results (p < 0.05).

**Table:** 

	**HADS-D **^**a**^ **≥11** **n=143** **n (%)**	**p** ^b^	**HADS-A**^**a**^ **≥11** **n=143** **n (%)**	**p** ^b^	**HADS-D**^a^ **or HADS-A** ^a^ **≥11** **n=145** **n (%)**	**p** ^b^
**All patients**	25 (17.4)		25 (17.4)		32 (22.1)	
**Male**	11 (7.6)	0.64	9 (6.3)	**<0.001**	13 (9.0)	0.12
**Female**	14 (9.7)		16 (11.2)		19 (13.1)	
**Age < 65**	24 (16.8)	0.85	23 (16.1)	0.18	30 (20.6)	0.58
**Age > 65**	1 (0.7)		2 (1.3)		2 (1.3)	
**Partnership**	18 (12.5)	0.23	19 (13.2)	0.26	24 (16.5)	0.63
**No partnership**	7 (4.8)		6 (4.1)		8 (5.5)	
**Children**	18 (12.5)	**0.023**	18 (12.5)	**0.044**	24 (16.5)	0.1
**No children**	7 (4.8)		7 (4.8)		8 (5.5)	
***Pre-existing psychiatric disorders***						
**Yes**	14 (9.7)	**<0.001**	14 (9.7)	**<0.001**	19 (13.1)	**0.003**
**No**	11 (7.6)		11 (7.6)		13 (8.9)	
***Ataractics***						
**Yes**	10 (6.9)	**<0.001**	9 (6.3)	**0.002**	13 (8.9)	**0.029**
**No**	15 (10.4)		16 (11.2)		19 (13.1)	

The authors would like to apologise for any inconvenience caused.

